# Platelet-Vesicles-Encapsulated RSL-3 Enable Anti-Angiogenesis and Induce Ferroptosis to Inhibit Pancreatic Cancer Progress

**DOI:** 10.3389/fendo.2022.865655

**Published:** 2022-03-24

**Authors:** Yiyin Zhang, Zhengze Huang, Jiaxi Cheng, Haoqi Pan, Tianyu Lin, Xuqiu Shen, Wenchao Chen, Qi Chen, Chenhui Gu, Qijiang Mao, Yuelong Liang

**Affiliations:** ^1^ Department of General Surgery, Sir Run Run Shaw Hospital, School of Medicine, Zhejiang University, Hangzhou, China; ^2^ Department of General Surgery, Hangzhou Fuyang Hospital of Traditional Chinese Medicine, Hangzhou, China; ^3^ Department of Orthopaedic Surgery, Sir Run Run Shaw Hospital, Medical College of Zhejiang University, Hangzhou, China

**Keywords:** pancreatic ductal adenocarcinoma, ferroptosis, RSL-3, anti-angiogenic, platelet vesicles

## Abstract

Pancreatic ductal adenocarcinoma (PDAC) is one of the most malignant cancers. It is characterized by stromal richness, lack of blood supply and special metabolic reprogramming in the tumor microenvironment, which is difficult to treat and easy to metastase. Great efforts have been made to develop new drugs which can pass through the stroma and are more effective than traditional chemotherapeutics, such as ferroptosis inducers–Erastin and RSL-3. As current anti-angiogenic therapy drugs alone are suboptimal for PDAC, novel vascular disruption agents in combination with ferroptosis inducers might provide a possible solution. Here, we designed human platelet vesicles (PVs) to camouflage RSL-3 to enhance drug uptake rate by tumor cells and circulation time *in vivo*, deteriorating the tumor vessels and resulting in tumor embolism to cut the nutrient supply as well as causing cell death due to excessive lipid peroxidation. The RSL-3@PVs can also cause the classic ferroptosis-related change of mitochondrial morphology, with changes in cellular redox levels. Besides that, RSL-3@PVs has been proved to have great biological safety profile *in vitro* and *in vivo*. This study demonstrates the promising potential of integrating PVs and RSL-3 as a combination therapy for improving the outcome of PDAC.

## Introduction

Pancreatic ductal adenocarcinoma (PDAC) is a malignant tumor originating from exocrine pancreatic ductal cells, accounting for 90% of all subtypes of pancreatic cancer (1). With worldwide increases in smoking, alcohol abuse, diabetes, chronic pancreatitis, and obesity, the new prevalence of PDAC is increasing yearly, and it is the 4^th^ leading cause of cancer-related death worldwide (2, 3). Surgical resection is currently the main treatment for PDAC, but due to the nonspecific symptoms of PDAC, most patients have already progressed to advanced stage when diagnosed (4, 5). Even after surgery, the prognosis is poor, and the 5-year survival rate is about 11% (1, 6). What’s more, the options for targeted therapy in PDAC are still very limited. PARP inhibitors can induce apoptosis by inhibiting DNA damage repair in tumor cells, which may be a useful therapeutic strategy for PDAC with *BRCA* mutations (7, 8). However, it is reported that only 8% of PDAC patients are harbored *BRCA* mutations. It indicates that the universality of PARP inhibitors is restricted, and some studies suggested that PARP inhibitors can cause significant side effects, such as anemia, nausea and leukopenia (9–11). Therefore, there is an urgent need for novel therapeutic strategies for PDAC to improve the dismal outcome.

Since there are six pathways to cause cell death, namely apoptosis, ferroptosis, necroptosis, autophagy, pyroptosis and necrosis. The basic research of ferroptosis has attracted much attention in recent years, and its important role in cancer treatment has also been widely appreciated (12). Ferroptosis is a kind of reactive oxygen species (ROS) -dependent cell death, which is characterized by the decrease or disappearance of mitochondrial cristae, the rupture of mitochondrial outer membrane and the condensation of mitochondrial membrane (13, 14). In the development and progression of malignant tumors, ferroptosis is considered to be an adaptive feature that eliminates cells that lack key substances or be damaged by environmental stress in a timely manner, thereby inhibiting tumorigenesis (15). For PDAC, previous studies have reported that some natural plant extracts, such as placentin A (CN-a), phenethyl isothiocyanate (16), artesunate (ART) (17), etc, can synergistically induce PDAC cell death through the production of ROS. The specific mechanism of ferroptosis in the progression of PDAC has not been clarified. In addition to the classical oxidative stress pathway, Zhu et al. found that heat shock protein family A (Hsp70) member 5 (HSPA5) regulates iron apoptosis in PDAC cells through HSPA5-GPX4 signaling pathway (18). Shintoku et al. suggested that lipoxygenase inhibitors inhibit the ferroptosis of pancreatic cells induced by Erastin and RSL-3 (19). In addition, NCOA4 is regarded as a cargo receptor mediating ferroptosis, and its silencing can suppress ferritin degradation and ferroptosis (20). At the same time, current studies tend to believe that ferroptosis is essentially a process of “iron accumulation-lipid peroxidation-cell membrane rupture”, which may lead to carcinogenic inflammatory injury, so ferroptosis may be a double-edged sword (21).

For the adjuvant therapy of PDAC after surgery, we noticed that the current single use of first-line chemotherapy gemcitabine in the treatment of PDAC has low response rate and often leads to drug resistance (22). According to the NCCN guidelines (Version 2021), the first-line treatments for PDAC, such as FOLFIRINOX, and nab-paclitaxel plus gemcitabine, are used as combination chemotherapeutic agents (23). Additionally, the complex interaction between cancer cells and tumor microenvironment (TME) plays an important role in tumor progression and treatment. As the “soil” of cancer cells, TME is conducive to promoting tumor growth, migration, invasion and angiogenesis (24–26). At present, various anti-angiogenic drugs are gradually being approved for cancer treatment, and some studies have reported that anti-angiogenic therapy may greatly improve the efficacy and long-term survival of PDAC patients (27, 28). For Erastin and RSL-3, Chen et al. believe that they can further reduce the malignancy of tumors and the risk of angiogenesis by inhibiting ATF4 (29). Based on that, we proposed to apply ferroptosis inducer combined with anti-angiogenesis therapy in the treatment of PDAC.

In recent years, the function of biological cell membranes for drug delivery *in vivo* has been continuously expanded (30), and the cell membranes of red blood cells, platelets, macrophages and cancer cells have been used to manufacture drug delivery carriers (31). Extracellular vesicles (EVs) are derived from the cell membrane, can also regulate the transport of substances and information exchange between many cells (32). EVs are convenient to extract and store with feature of economic friendly (33). In addition, platelets are regarded as important members of immune action *in vivo*, contribute to the initiation and coordination of intravascular immune response, and participate in the regulation of tumor occurrence and development (34). Encapsulation of platelet membrane (PMs) or platelet vesicles (PVs) is an emerging approach of drug delivery. Glycoprotein receptors, cell adhesion molecules and other components on the surface of platelets endow platelets with the ability to interact with a variety of pathogenic biological substrates. Autoantigens such as CD47 can evade the clearance of the immune system, prolong the circulation time in the body and promote the accumulation of drugs in the focus, thus avoiding the embarrassment of rapid liver uptake and reduction of plasma concentration determination as soon as the drugs are in the circulation system (35). At the same time, PV-coated technology may have the ability to regulate ferroptosis response. Jiang et al. prepared PV-coated magnetic nanoparticles and found that they could repolarize macrophages with immunosuppressive M2 phenotype into anti-tumor M1 phenotype, and by inhibiting the systemic cysteine/glutamate transporter pathway, inducing the occurrence of ferroptosis in tumor cells (36). On the other hand, the massive aggregation of PVs at the tumor site is conducive to the formation of tumor vascular embolism (37), which inhibits the initial angiogenesis and the expansion of the tumor neovascular network, and prevents the further development of the tumor (38). Therefore, PVs not only endows the encapsulated drugs with tumor-targeting ability and biocompatibility, but also increases their accumulation in the tumor site by targeting and adhering to damaged blood vessels through surface receptors, which has great advantages in clinical application and developmental prospects compared with single drug.

**Scheme 1:**
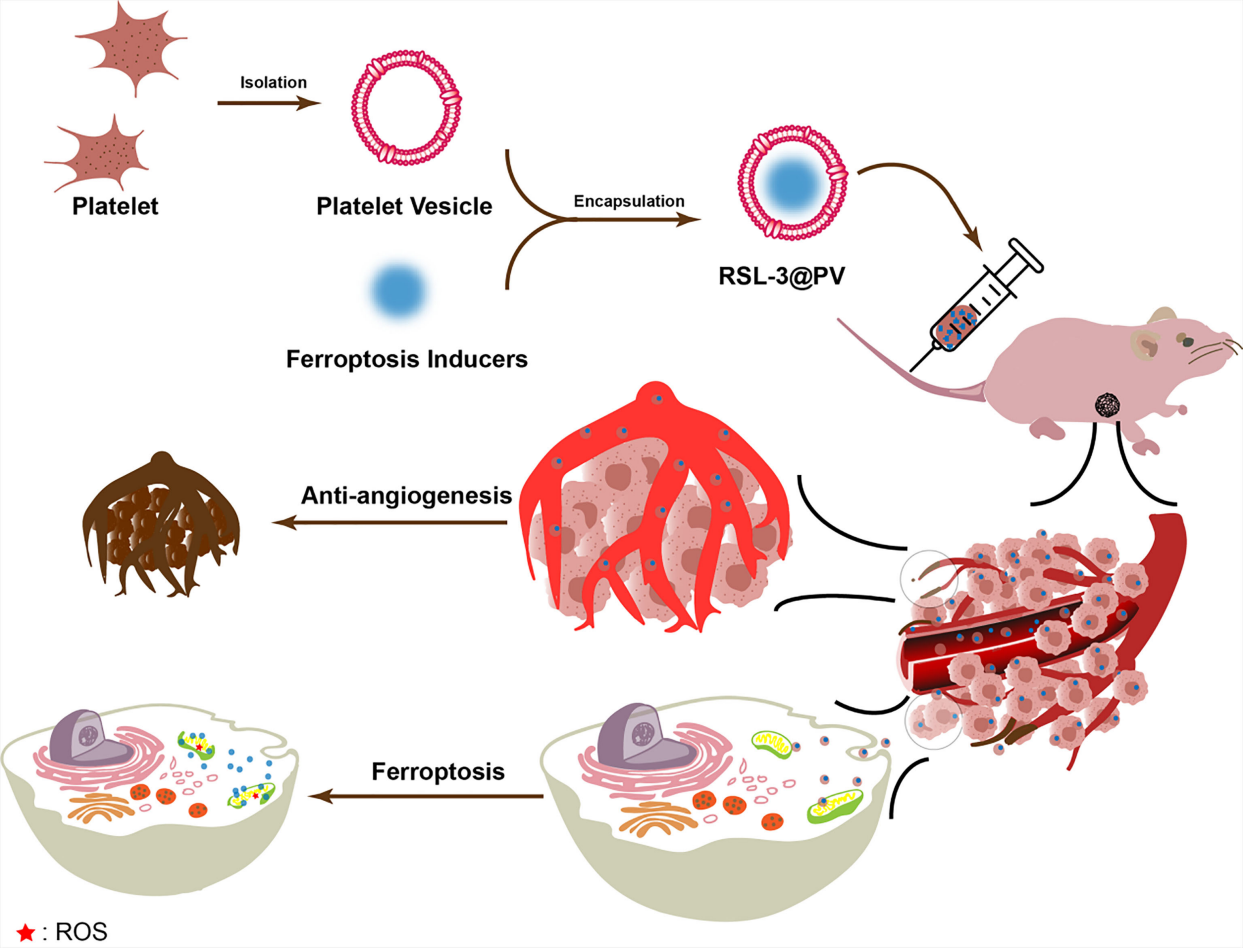
Schematic representation of the inhibitory mechanism of RSL-3@PVs on pancreatic cancer cells.

In our study, as showed in **Scheme 1**, RSL-3@PVs, a PV-encapsulated ferroptosis inducer, was applied to treat PDAC, and its long-term inhibitory effect on PDAC cells was verified *in vivo* and *in vitro*. We characterized the material properties of RSL-3@PVs and validated its excellent targeting ability and biosafety *in vitro*. Finally, RSL-3@PVs was injected into the tail vein of mice with subcutaneous tumor of PDAC, and the tumor volume, mass, inflammatory indicators and thrombosis were monitored, which further verified the satisfactory efficacy of RSL-3@PVs *in vivo*. To sum up, RSL-3@PVs can give full play to the inhibitory effect of ferroptosis on PDAC cells, combined with the superior targeting ability and biocompatibility of PVs to effectively form vascular embolization of tumor cells, thus achieving an ideal inhibitory effect on the growth of PDAC cells, with the effect of one plus one is better than two. It has a broad application prospect in the combination treatment of PDAC in the future.

## Materials and Methods

### Preparation of PVs and RSL-3@PVs

Platelet plasma was isolated from the peripheral blood of human by a two-step centrifugation. Then the platelet plasma was centrifuged (800 g, 20 min, 4°C), after discarding the supernatant, the pellet was resuspended in PBS buffer, and was centrifuged again to obtain a purified platelet solution that was stored at -20°C. PVs were obtained by means of extrusion. The RSL-3 solution was mixed with purified platelet solution at 4°C and incubated for four hours and RSL-3@PVs were obtained by extrusion.

### Cell Culture

The pancreatic cancer cell lines PANC-1, PANC-2 and MIA PaCa-2 were purchased from the American Type Culture Collection and verified by DNA fingerprinting. Cells were cultured in DMEM containing 10% FBS in a 5% CO_2_ incubator at 37°C (Thermo, USA).

### Characterization

Transmission electron microscopy (TEM) images were obtained by a Tecnai G2 Spirit 120 kV TEM (FEI, USA) and Talos 120kV cryo TEM (Thermo). The DLS and zeta potential values were measured on a Malvern Zeta-sizer Nano instrument (Malvern, UK), and the absorption spectra were recorded using UV-2000 photo-spectrometer with UV-Probe v2.42 (Shimadzu, Japan).

### Quantitative Real-Time PCR (qRT-PCR) Detection and Quantitative Analysis

RNA-Quick Purification Kit (ES Science, China) was used to isolated the total RNA, absorption measurements were using to assess quality and quantity. RNA was then reverse transcribed into cDNA using an Evo M-MLV RT Premix kit (Accurate Biology, China). The cDNA was added with Hifair™ qPCR SYBR Green Master Mix (Yeasen, China) to form a mixture, then the expression levels of the candidate genes were determined by ABI 7900HT Real-Time PCR system (Applied Biosystems, USA). Through PCR Array Data analysis web portal, the 2^-ΔΔCt^ method was used for comparative data analysis (http://gncpro.sabiosciences.com/gncpro/gncpro.php) to determine relative expression differences between the comparison groups. All reactions were run in triplicate, and primer sequences are listed in **Supplementary Table**.

### *In Vitro* Cytotoxicity Assays

PANC-1, PANC-2 and MIA PaCa-2 cells were incubated in 96-well plates (5 × 10^3^ cells per well with 100 μL of suspension) for 12 hours. Then, the culture medium was replaced with 100 μL fresh culture medium containing PVs, RSL-3 or RSL-3@PVs at different concentrations. The cells were cultured for another 24 hours, and the cell viability was evaluated by the MTS assay (Promega, USA). After the culture medium was removed, PBS was added and the wells were washed three times, and fresh medium containing 10% MTS reagent was added and incubated for another three hours. Multi detection microplate reader (Thermo) was used to detect the absorbance at 490 nm and evaluated the cell viability. The relative cell viability was calculated using the following formula: relative cell viability (%) = (mean experimental absorbance/mean control absorbance) × 100%.

### Establishment of the Tumor Xenograft Model

Four-week old BALB/c-nu male mice were housed in sterile and filter capped cages for one week prior to subcutaneous tumor implantation. Mice were randomly divided into four groups (n = four/group) and injected with a solution containing 3 × 10^6^ PANC-2-Luc cells to establish the PANC-2-Luc tumor models. After tumor formation, 200 μL of PBS, PVs (4.41mg/ml), RSL-3 (4.41mg/ml) or RSL-3@PVs (4.41mg/ml) solution was injected into the tail vein every other day for three consecutive times. Xenograft sizes were measured three times a week and volume were determined using the following formula: length × width^2^/2. The study was approved by the Animal Use and Care Committee at Zhejiang University.

### Immunohistochemistry (IHC)

Tissue paraffin sections were dewaxed in xylene and rehydrated in ethanol. The slides were immersed in a 10% (w/v) sodium citrate solution at 95°C for 30 min (antigen retrieval) and then cooled to room temperature. Endogenous peroxidase activity was blocked with 3% (v/v) hydrogen peroxide and BSA added to 3% (w/v) followed by incubation at room temperature for 30 min. Tissue slices were next incubated with primary antibodies, Ki-67 (1:2000, Proteintech, USA), anti-4-Hydroxynonenal (4-HNE, 1:100, Abcam, UK) and anti-CD31 (1:100, Abcam) at 4°C overnight, the secondary antibodies (1: 1000) were then added for incubation for one hour. The sections were dehydrated after color development with 3,3′-diaminobenzidine and counterstaining with hematoxylin. Finally, protein expression was observed under a microscope (Leica Microsystems, Germany). The immunohistochemical staining score was obtained by multiplying the percentage of staining positive cells, 0 represents for less than 5%; 1 represents for 5-25%; 2 represents for 25-50%; 3 represents for 50-75% and 4 represents for more than 75%. The staining intensity, 0 represents for negative; 1 represents for weak; 2 represents for moderate and 3 represents for strong. The expression levels were classified as follows: negative represents for 0, -; weak represents for 1-3, +; moderate represents for 4-6, ++ and strong represents for more than 6, +++.

### *In Vivo* Biosafety of RSL-3@PVs

ICR mice were intravenously injected with 200 μL PBS, PVs (4.41 mg/ml), RSL-3 (4.41 mg/ml) or RSL-3@PVs (4.41mg/ml), three ICR mice in each group. After 24 hours, all the mice were sacrificed. Blood samples were collected by eyeball extraction, and hematology studies were performed. Blood biochemistry data of liver and kidney function markers including alanine transaminase (ALT), aspartate transaminase (AST), and blood urea nitrogen (BUN) (n = 3). The vital organs (brain, heart, liver, spleen, lung, and kidney) were surgically dissected, prior to being fixed with 4% paraformaldehyde for 24 hours, embedded in paraffin, sliced, as well as stained with hematoxylin and eosin (H&E) according to the manufacturer’s manual, and observed under an inverted optical microscope (Zeiss, Germany).

### *In Vivo* Fluorescence (FL)

For *in vivo* FL imaging, the PANC-2-Luc-bearing mice were intravenously injected with D-luciferin potassium salt (10 μL, 10 mg/mL) *via* the tail vein. FL imaging (Ex: 640 nm and Em: 720 nm) was performed at 15 min post injection using an *in vivo* imaging system (PerkinElmer, USA). After FL scanning at the baseline and at day 21, the mice were euthanized and all the tumor specimens were harvested and fixated with 4% paraformaldehyde before being sectioned into tissue slices for IHC and H&E.

### *In Vitro* Subcellular Localization of RSL-3@PVs

To determine the cellular uptake and subcellular localization of RSL-3@PVs. PANC-1, PANC-2 and MIA PaCa-2 cells were seeded on 24-plate dishes and incubated 20 min with the DiI (C1995S, Beyotime, China)-labelled RSL-3@PVs at 37°C. After gentle washing with PBS, the nuclei were counterstained with Hoechst 33258 (C1011, Beyotime) for 15 min at room temperature The subcellular localization of RSL-3@PVs was examined using a fluorescence microscope (Zeiss) at an excitation wavelength of 560 ± 20 nm and an emission wavelength of 650 ± 5 nm for DiI, an excitation wavelength of 360 ± 20 nm and an emission wavelength of 460 ± 25 nm for Hoechst.

### Glutathione Reductase (GR) Assay and Lipid Peroxidation Malondialdehyde (MDA) Assay

The determination of GR and MDA were practiced as the instruction of the manuals of Lipid Peroxidation MDA Assay Kit (S0131S, Beyotime) and GR assay kit (S0059S, Beyotime).

### Western Blot

Western blot was performed as previously described (39). The antibodies used in the current study were anti-ALIX antibodies (92880, Cell Signaling Technology, USA), anti-GPX4 antibody (ab41787, Abcam), anti-FTH1 antibody (3998, Cell Signaling Technology), anti-KEAP1 antibody (8047, Cell Signaling Technology), anti-SLC7A11 antibody (12691, Cell Signaling Technology), anti-CD31-antibody (11265-1-AP, Proteintech) and anti-α Tubulin antibody (11224-1-AP, Proteintech).

### Statistics

Graphpad prism (version 8.0, USA) was used for the data analysis and Student’s *t*-tests were applied for most occasions. Experiments were repeated at least three times and were presented as the mean ± SD. One-way ANOVA was used to analyze the differences among three or more groups. Statistical differences were regarded significant at *, *P < 0.05; **P < 0.01, and ***P < 0.001.

## Results

### Preparation and Characterization of RSL-3@PVs

**Figure 1:**
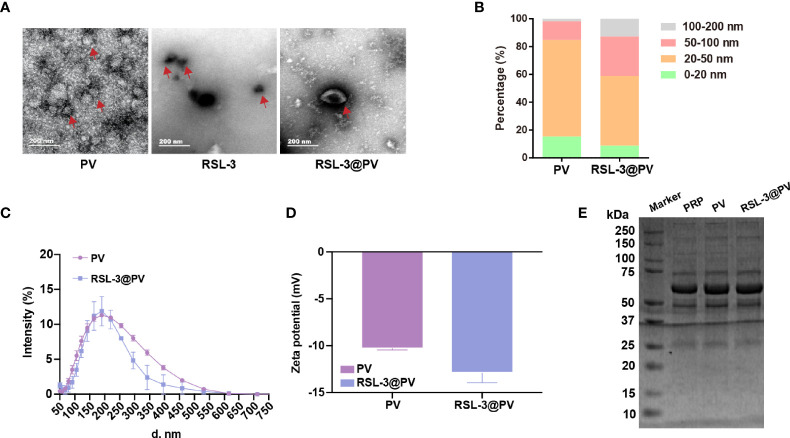
Characterization of RSL-3@PVs. **(A)** TEM images of PV, RSL-3, and RSL-3@PVs. Scale bars: 200 nm. **(B, C)** Hydrodynamic diameters distribution of PV and RSL-3@PVs. **(D)** Zeta potentials of PV and RSL-3@PVs. **(E)** SDS-PAGE analysis of the platelet-related proteins in PRP, PV, and RSL-3@PVs.

RSL-3 were commercially purchased from TOPSCIENCE (CAS 1219810-16-8, China), which was dissolved in DMSO. The spherical morphology of the PVs, RSL-3s and PVs coated RSL-3s was verified by TEM (**Figure 1A**). We made a statistical analysis to distinguish the size of different particles under the TEM. According to our calculation, PVs were mainly concentrated in 20-50 nm while with RSL-3 inside, the RSL-3@PVs were mainly concentrated in 20-50 nm and 50-100 nm (**Figure 1B**). **Figure 1C** showed that the diameters of PVs and RSL-3@PVs were 197.08 ± 4.89 and 153.29 ± 14.42 nm, indicating that after the vesicles wrap up the drug, they would further tighten, and reduce their size. Also, coating RSL-3s with PVs led to a slight decrease in zeta potential from -10.3 ± 0.14 mV to -12.9 ± 1.04 mV (**Figure 1D**). To make sure that the coating process would not affect the properties of PV proteins, the platelet-riched plasma (PRP), PVs and RSL-3@PVs were lysed, and the supernatants were later performed SDS-PAGE analysis. **Figure 1E** showed that the protein profile of PRP was similar to PVs and RSL-3@PVs, suggesting that complete reservation of vesicle proteins of RSL-3@PVs.

### *In Vitro* Cell Targeting Ability of RSL-3@PVs

**Figure 2:**
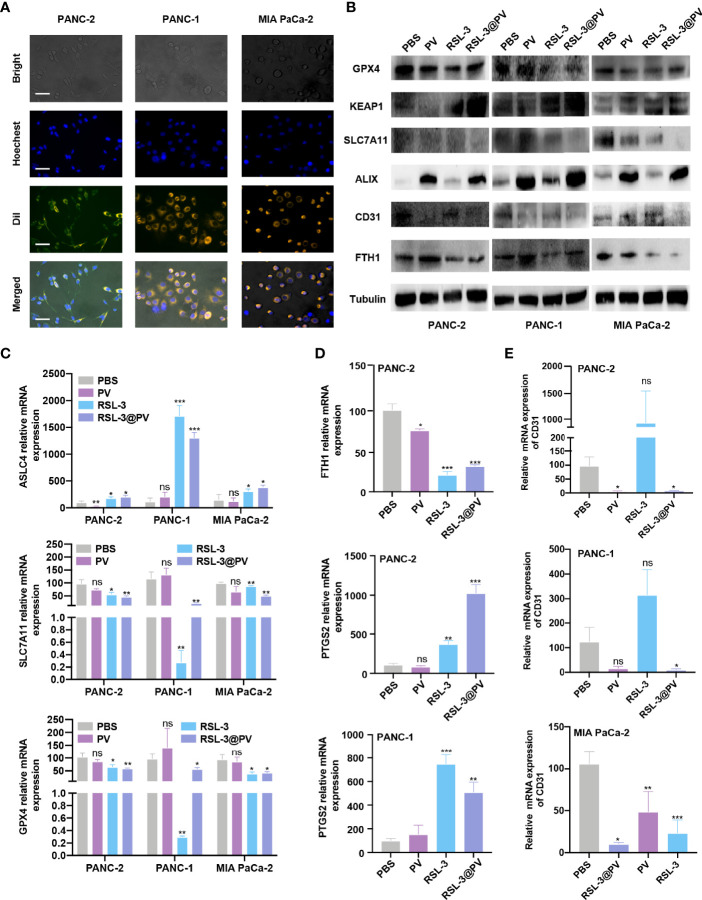
*In vitro* cell targeting of RSL-3@PVs. **(A)** Inverted optical microscope images of PANC-2, PANC-1, MIA PaCa-2 treated with RSL-3@PVs. Cell nuclei and membranes were labeled with DAPI and DiI, respectively. Scale bars: 200 μm. **(B)** Western blot analysis of the ferroptosis core regulators and vesicle marker in PANC-2, PANC-1, MIA PaCa-2 treated with PBS, PV, RSL-3 and RSL-3@PVs. **(C–E)** Relative mRNA expression of *ASLC4*, *SLC7A11*, *GPX4* **(C)**, *FTH1*, *PTGS2* **(D)**, *CD31* **(E)**. Error bars represent the mean ± s.d. One-way ANOVA was used for multiple comparisons, ns P > 0.05, *P < 0.05, **P < 0.01, and ***P < 0.001.

**Figure 3:**
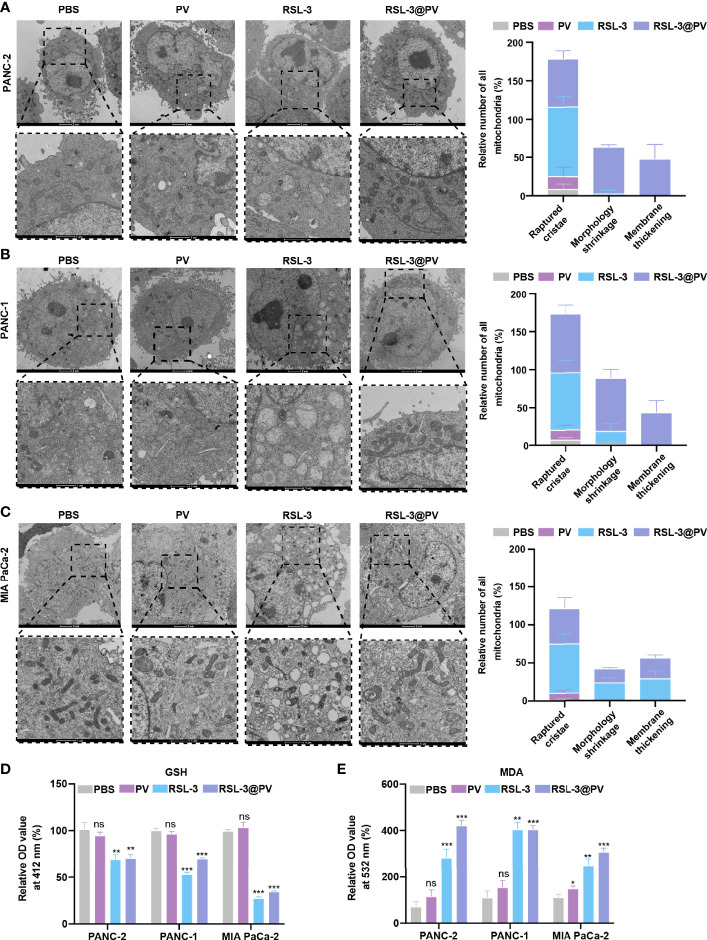
Effects of ferroptosis on cell morphology and levels of oxidative stress. **(A–C)** TEM images and relative number of all mitochondria in PANC-2 **(A)**, PANC-1 **(B)**, MIA PaCa-2 **(C)** treated with PBS, PV, RSL-3 and RSL-3@PVs. Scale bars: 2 μm and 1 μm. **(D)** Relative OD= 412 nm value in PANC-2, PANC-1, MIA PaCa-2 treated with PBS, PV, RSL-3 and RSL-3@PVs, which characterize the GSH levels. **(E)** Relative OD = 532 nm value in PANC-2, PANC-1, MIA PaCa-2 treated with PBS, PV, RSL-3 and RSL-3@PVs, which characterize the MDA levels. Error bars represent the mean ± s.d. One-way ANOVA was used for multiple comparisons, ns P >0.05, *P < 0.05, **P < 0.01, and ***P < 0.001.

To analyze whether PVs have the ability to target tumor cells, we incubated two human- and one murine- derived pancreatic cancer cell lines for three hours, with DiI-label RSL-3@PVs. The cell nuclei were stained with Hoechst and scanned by inverted fluorescence microscope. It could be seen that a significant red fluorescence (DiI) was presented around the cytomembrane in three cell lines, indicating that RSL-3@PVs has good cell targeting *in vitro* (**Figure 2A**). To validate if the vesicles would affect the ability of RSL-3 to induce ferroptosis and maintain its ability of anti-angiogenesis, we first evaluated the IC_50_ of RSL-3 in three cell lines (**Supplementary Figure 1** and **Figure 2**, **3**). Then we performed western blotting to clarify the expression of related indicators at the protein level. As shown in **Figure 2B**, ferroptosis core regulators GPX4 and SLC7A11, as well as FTH1 were down-regulated in RSL-3s and RSL-3@PVs groups compared with PBS and PVs, while KEAP1 was upregulated after being treated with RSL-3s and RSL-3@PVs. The characteristic of PVs maintained after encapsulating RSL-3, as the vesicle marker ALIX expressed similarly to that of PVs. Also, the property of angiogenesis inhibition was not changed after combining with RSL-3, as the expression of CD31 remained the same level in RSL-3@PVs as in PVs. We further performed qPCR to clarify the expression of the related indicators at mRNA level. We found that the expression of *ASLC4* was upregulated in RSL-3s and RSL-3@PVs in PANC-2, PANC-1 and MIA PaCa-2 (**Figure 2C**). Similar to the expression changes at protein level, the relative mRNA expression of *GPX4* and *SLC7A11* were downregulated in RSL-3s and RSL-3@PVs in comparison with PBS and PVs. Additionally, the relative mRNA expression of *PTGS2* was elevated in RSL-3s and RSL-3@PVs compared with the control groups in PANC-2; it was also observed that the expression of *PTGS2* was relatively lower in PBS and PVs compared with RSL-3s and RSL-3@PVs (**Figure 2D**). The CD31 expression was involved in leukocyte migration, angiogenesis and integrin stimulation. The angiogenesis level of PVs decreased significantly, which was the same as that of RSL-3@PVs in PANC-2 and MIA PaCa-2 (**Figure 2E**; PANC-2, both P < 0.05; MIA PaCa-2, P < 0.05 and P < 0.001). Even though the expression of CD31 was not statistically significant in PANC-1 in PVs, its downward trend was obvious (P = 0.0588).

### Morphological and Peroxidation Level Changes in Cells and After Ferroptosis Induction

In order to further investigate whether the addition of the ferroptosis inducer RSL-3 would affect the response of pancreatic cancer cells during ferroptosis, and whether the encapsulation of RSL-3 with PVs will affect the induction of ferroptosis, we decided to use TEM to observe the morphological changes inside the cancer cells. As shown in **Figures 3A–C**, the cell membranes ruptured and blebbed during ferroptosis, with mitochondria atrophied, mitochondrial ridges decreased or even disappeared; the morphology of nucleus was normal, but there was chromatin condensation; the intracellular mitochondria became smaller and membrane density increased in RSL-3s and RSL-3@PVs compared with PBS and PVs. The quantitation of mitochondria number remained alike between the four groups in three cell lines while the raptured cristae, morphology shrinkage and membrane thickening increased after the addition of RSL-3.

GSH/GSSH is one of the most important redox pairs in cells. Therefore, measuring the content and ratio of GSH and GSSG in cells can reflect the redox state of cells. As shown in **Figure 3D**, the GSH content descended rapidly after the addition of RSL-3s and RSL-3@PVs. MDA is a marker of ferroptosis because it is one of the products of cell membrane lipid peroxidation. It is usually used as an indicator of lipid peroxidation to indicate the degree of lipid peroxidation of cell membrane. As shown in **Figure 3E**, the MDA level elevated after the treatment with RSL-3s and RSL-3@PVs. These results indicated that the encapsulation of PVs did not affect the function of RSL-3.

### *In Vivo* Tumor Killing Ability of RSL-3@PVs

**Figure 4:**
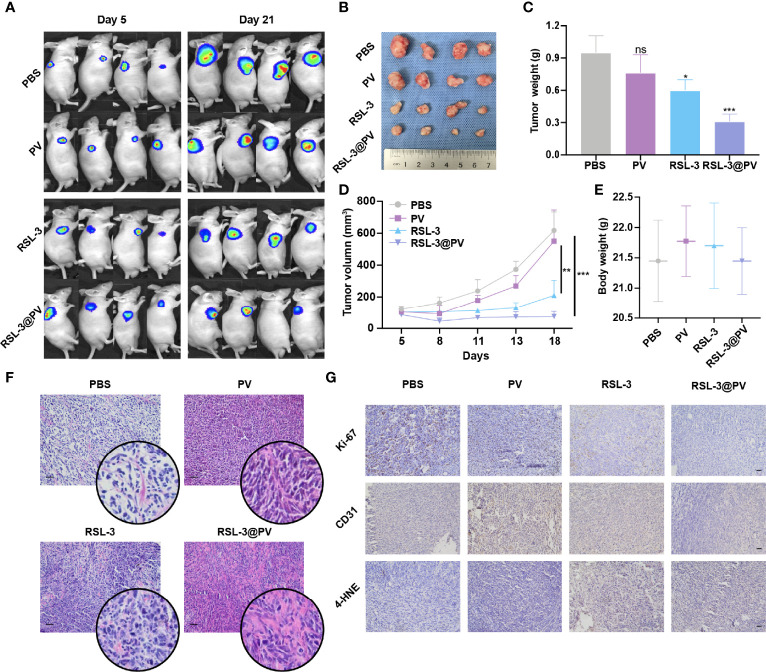
Antitumor activity of RSL-3@PVs. **(A)** Fluorescence images of tumors at day 5 and day 21 after injection of the indicated formulations into PDAC xenograft nude mice (n = 4). **(B)** Representative images of tumors excised at the end of the experiment (n = 4). **(C)** The volumes of tumors collected at the end of treatment (n = 4). **(D)** Tumor growth curves of PDAC xenograft BALB/c nude mice treated with the indicated formulations (n = 4). **(E)** The body weights of mice measured at the end of treatment (n = 4). **(F)** H&E staining of the dissected tumor tissues in the indicated treatment group. Scale bars, 100 μm. **(G)** Ki-67, CD31 and 4-HNE staining of the dissected tumor tissues in the indicated treatment group. Scale bars, 100 μm. Error bars represent the mean ± s.d. Student’s *t* test or one-way ANOVA was used for comparison between two groups or among multiple groups, ns P > 0.05, *P < 0.05, **P < 0.01, and ***P < 0.001.

RSL-3@PVs were next evaluated for the antitumor ability in the xenograft model of PDAC on the right scapular of BALB/c nude mice. We first detected the baseline of the PANC-2-Luc tumors in four groups containing 16 mice (**Figure 4A**). After measuring the baseline tumor level of approximately 100-200 mm^3^, we injected four different drugs into the tail vein of mice on three consecutive alternate days and measured them two times a week. On day 21, all mice were sacrificed to remove the tumors. Similar to imaging effect in **Figure 4A**, the tumor sizes diminished significantly after using RSL-3 and even more remarkably after using RSL-3@PVs (**Figure 4B**). The PBS group had twice the tumor weight than in the RSL-3@PVs group (P < 0.001; **Figure 4C**). Compared with the PBS group, the tumor volume of RSL-3s (P < 0.05) and RSL-3@PVs groups increased relatively slowly, especially in the RSL-3@PVs group, the baseline tumor level of approximately 100-200 mm^3^ maintained (P < 0.001; **Figure 4D**). There was little difference in weight change between groups (**Figure 4E**). To investigate the underlying mechanism in the tumor killing effect of RSL-3@PVs, we performed H&E staining in the tumor sections. Apart from more apoptosis and necrosis in the control group, we found that there were more tumor embolisms in the RSL-3@PVs compared with other three groups (**Figure 4F**). It was possible that PVs could inhibit tumor growth by affecting tumor blood supply. To further validate our hypothesis, we stained CD31 on the tumor slices to assess vessel density. RSL-3@PVs exhibited the excellent tumor vascular disruption and inhibition of tumor neovascularization as the vascular density decreased (**Figure 4G**). Moreover, 4-HNE– an α, β unsaturated hydroxyalkenal, is an oxidative/nitrosative stress biomarker. When ferroptosis increases, lipid peroxides increase, and 4-HNE expression also increases. We found that the protein level expression of 4-HNE was relatively higher in RSL-3s and RSL-3@PVs compared with PBS and PVs (**Figure 4G**). Under the combined treatment of the two drugs, we found that the proliferation ability of PDAC cells was significantly decreased, as the Ki-67 decreased rapidly in the RSL-3s and RSL-3@PVs (**Figure 4G**). These results indicated that the PVs and RSL-3s might have synergistic effects in cutting the nutritional supply of PDAC and causing tumor death *via* ferroptosis.

### *In Vitro* and *In Vivo* Safety Evaluation

**Figure 5:**
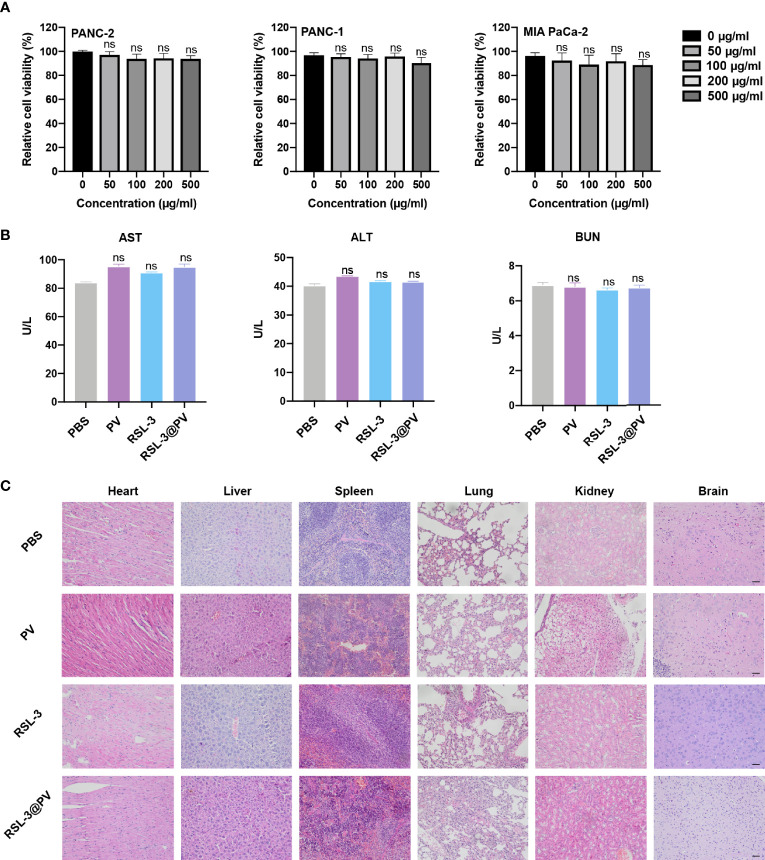
*In vitro* and *in vivo* safety evaluation of RSL-3@PVs. **(A)** Relative cell viability of PANC-2, PANC-1, MIA PaCa-2 at different concentrations. **(B)** Liver and kidney function (AST, ALT and BUN) in serum of healthy ICR mice after treatment with the indicated formulations. **(C)** Representative images of major organs from mice after treatment with the indicated formulations. Scale bars, 100 μm. Error bars represent the mean ± s.d. One-way ANOVA was used for multiple comparisons, ns P > 0.05.

To investigate whether RSL-3@PVs had fine biosafety profile *in vitro*, we added the PBS and PVs, to the three cancer cell lines and incubated them for 24 hours to observe the difference of cell survival rate between each group. As shown in **Figure 5A**, there were no significant difference in cell survival between 0, 50, 100, 200 and 500 ug/mL PV concentrations, suggesting that PVs itself have a good biocompatibility. We next investigated the biocompatibility of the RSL-3@PVs at the current treatment dosage *in vivo*. No apparent changes and differences in body weight of the mice were found in any group during the entire therapeutic process (**Figure 4E**). We then evaluated the liver and kidney function (ALT, AST and BUN) in serum of the healthy ICR mice divided in four groups. At the same time, we removed the heart, liver, spleen, lung, kidney, and brain of all mice 24 hours after tail vein injection of drugs. There were no apparent changes in liver and kidney function between groups (**Figure 5B**). H&E staining also did not indicate any structural damage in any of the major organs (**Figure 5C**). All these results revealed a good biosafety profile of RSL-3@PVs.

## Discussions

In this study, we present the first attempt to treat PDAC with the human PV-encapsulated ferroptosis inducer RSL-3 as a combination therapy. We demonstrated that PV-encapsulated RSL-3 not only fused with the property of PVs to embolize tumors *in vitro*, but also did not interfere with the property of RSL-3 to cause ferroptosis of tumors. We also observed mitochondrial atrophy, reduction or even disappearance of mitochondrial cristae, increase of membrane density and normal nuclear morphological changes in cell structure. Lipid peroxidation was found in PDAC cells treated with RSL-3 and RSL-3@PVs *via* measurement of GSH and MDA *in vivo*. In the mouse xenograft tumor model, after the treatment of RSL-3@PVs, the tumor of RSL-3@PVs maintained the baseline size compared with the other three groups, achieving a good therapeutic effect. This points out that RSL-3@PVs as a combination therapy, it has more efficacy advantages than PVs and RSL-3s monotherapy, and it is a safe treatment with high biocompatibility, which not only comes from human platelets, but also returns to human body, which provides a new hope for combination targeted therapies for PDAC in the near future.

For PDAC, as one of the most malignant tumors with the highest mortality rate, chemotherapy is still the mainstream therapy when the disease progresses to the point that surgery cannot be performed (40). Targeted therapy and immunotherapy have also made some breakthroughs in recent years (41). However, patients with PDAC usually have a low tumor mutation burden (TMB), and a high TMB is often positively correlated with the therapeutic efficacy of immune checkpoint inhibitors (ICIs). Clinical studies have shown that the overall response rate (ORR) of the combination therapy of pabolizumab, gemcitabine, nab-paclitaxel is 18.2% (42). Given that different mismatch repair (dMMR) tumors are characterized by a high TMB and that dMMR is currently presented in 1% of patients with PDAC, pabolizumab is still recommended as a second-line therapy. At the same time, a large number of studies are expected to ameliorate the efficacy of PDAC immunotherapy by improving T cell infiltrations (43), enhancing antigen exposures (44), or exploring novel potential therapeutic targets (45), but in view of the fact that PDAC is a tumor with extremely rich stroma, it determines the particularity of its immune inactivity. In addition to the specificity of its immune environment, 90% of PDAC patients were harbored untargetable *KRAS* mutation, which plays a leading role in metabolic reprogramming to promote the growth and metastasis of PDAC. In this process, a large amount of ROS would be produced, and PDAC cells would consume abundant cysteine to resist oxidation, that is, glutathione. As PDAC is cysteine-dependent, the inhibition of cysteine can cut the supply of nutrients, thus changing the metabolic reprogramming of PDAC. Therefore, using the immune and metabolic characteristics of PDAC to focus on ferroptosis-related drugs can yet be regarded as a good way to break through the limitations of traditional therapies. At present, more and more studies have focused on the cell death pathways of PDAC, including apoptosis and ferroptosis. It has been found that ferroptosis is related to metabolic events such as lipid synthesis, autophagy and mitochondrial TCA cycle. Meanwhile, the E-cadherin-NF2-Hippo-Yap pathway and glucose-regulated AMPK signaling pathway (46) are also involved in regulation of metabolism. In the future, it is expected to discover more cell death pathways and targets, to overcome the dilemma of PDAC treatment.

PVs have a near-similar composition to platelets so that are implicated in many vascular injury-related diseases, such as cancer, inflammation, and trauma. Therefore, PVs are regarded as potential targets for the treatment of varieties of diseases. Numerous studies have found that PVs inhibit tumor tissue in mouse models of colorectal cancer (47), liver cancer (48), breast cancer (49), and ovarian cancer (50). It has been reported that nanoparticles synthesized after PV encapsulation of doxorubicin and vancomycin showed natural affinity for breast cancer cells, and could assist inhibiting the progression of breast cancer (51). More importantly, some studies have found that platelets could be enriched in defective tumor blood vessels *in vivo*, further secrete nanoscale PVs under the activation of photothermal or immune agonists, thus eliminating more tumor cells and enhancing the immunogenicity of tumor antigens (33). At the same time, PVs have also been shown to have advantages in the treatment of vascular diseases such as atherosclerosis, myocardial infarction and *Staphylococcus aureus* infection (52, 53). *In vitro*, we found that RSL-3@PVs had a good ability to target tumor cells. Moreover, for tumors, “self-markers” such as CD47 presented on PVs allow them to evade immune clearance, thereby prolonging circulation time and preferentially accumulating at tumor sites through enhanced permeability and retention effect (54). This can alleviate the problems that many nanomaterials are easily ingested by the liver when they enter the body, resulting in low blood concentration, short circulation time, and poor efficacy. On the other hand, some studies suggested that platelets can also bind to individual Circulating Tumor Cells (CTCs) that break away from the basement membrane and enter the blood circulation while exerting immune function, thus promoting tumor metastasis (55). It is possible that PVs-coated with RSL-3 may be able to bind to CTCs in time and induce ferroptosis, which may play an indispensable role in suppressing the further metastasis of CTCs and provide the new mentality for diagnosis and treatment for PDAC.

In the meantime, we closely followed the inhibitory effect of ferroptosis on PDAC cells. Ferroptosis is essentially a process of “iron accumulation-lipid peroxidation-cell plasma membrane rupture” (21). On the one hand, we found that RSL-3 can increase the expression of 4-HNE, a biomarker of lipid peroxide level, which also means that RSL-3 induces oxidative stress. GSSG/GSH is one of the most important redox couples in cells, and the increase of its ratio is often used as a marker of oxidative stress (56). Our results showed that both RSL-3 and RSL-3@PVs could reduce the level of GSH, indicating that RSL-3 could induce PDAC cells to experience a higher level of oxidative stress. The end product of lipid peroxidation in organism is MDA, which can cause the cross-linking polymerization of life macromolecules, such as protein and nucleic acid, and has cytotoxicity. Its content is an important parameter reflecting the potential antioxidant capacity of the body, which can reflect the rate and intensity of lipid peroxidation, and also indirectly reflect the degree of tissue peroxidation damage. RSL-3 and RSL-3@PVs increased MDA levels compared to PBS and PVs. On the other hand, through TEM observation, we found that RSL-3 and RSL-3@PVs could induce cell membrane rupture to different degrees, mitochondrial atrophy, mitochondrial cristae reduction or even disappearance, which verified that RSL-3 and RSL-3@PVs could induce the iron death process of PDAC cells by damaging mitochondrial function (57).

Angiogenesis is the most essential factor for tumor growth and metastasis. As a “transport channel”, neovascularization of tumor provide abundant oxygen and nutrients for the rapid proliferation of cancer cells (58). Whether it is an early staged or end staged cancer, or even a residual cancer cell after intensive anti-cancer treatment, it must rely on similar blood supply to continue growing. On the other hand, the extensive neovascularization in the local or surrounding tumor is also the key “channel” for cancer cells to enter the blood and metastasize, so that free cancer cells can quickly spread to distant places with systemic blood flow (59). The core mechanism of anti-angiogenesis therapy is to destroy or suppress the local new blood vessels of tumors, cut off the oxygen and other nutrients needed for the growth of tumor cells, and destroy the blood metastasis pathway of cancer cells, which is vividly called “tumor starvation therapy”. Previous studies have suggested that the “avascular” characteristics of PDAC, that is, the lack of sufficient new blood vessels in the tumor, may be an important reason for the poor anti-angiogenic efficacy (60). Subsequent studies have confirmed that when the vascular integrity of PDAC is poor and the tumor with low microvascular density can still maintain a high frequency of tumor hematogenous metastasis; only when the microvascular density is high and the vascular integrity is poor, the chance for tumor metastasis is the greatest (61). In the study, we used xenograft mouse model to characterize the PDAC inhibitory effect of RSL-3@PVs. Our findings showed that RSL-3@PVs could effectively inhibit the progression of tumor mass and volume. In order to further explore the specific process of RSL-3@PVs participating in tumor killing, we observed the blood supply of the tumor, and found that there was extensive vascular embolism in the tumor tissue under the action of RSL-3@PVs, and the vascular density was greatly reduced, thus achieving a large area of hypoxia and necrosis of PDAC tissue around the damaged blood vessels (62). Therefore, we guess that embolization of blood vessels is an alternative way for anti-angiogenic treatment if it cannot improve the integrity of blood vessels. Anti-angiogenic therapy and immunotherapy are the main treatment strategy aiming at TME at present (63), and their combined application can control and kill tumor cells synergistically. Recently, VEGFRs peptide vaccines have been considered to hold the advantages of anti-angiogenic therapy and immunotherapy and have been extensively studied and tested in patients with progressive neurofibromatosis type 2 (NF2) (64), which also implies the potential of anti-angiogenic therapy in combination with other drugs in cancer therapy. Therefore, in this research, we also attempt to apply RSL-3@PVs as the combination therapy for PDAC, and the results were satisfied.

Remarkably, we demonstrated that the combination of human PVs and RSL-3 did not inhibit the therapeutic effect of any component but promoted the synergistic inhibition of PDAC progression *via* both ferroptosis and tumor embolism *in vitro* and *in vivo*. Moreover, we also verified the biological safety of RSL-3@PVs and found that it did not cause structural damage to any other major organs except tumors, which further proved the biological safety of RSL-3@PVs and provided a practical experimental basis for its clinical transformation and wide application.

## Data Availability Statement

The datasets presented in this study can be found in online repositories. The names of the repository/repositories and accession number(s) can be found in the article/**Supplementary Material**.

## Ethics Statement

The animal study was reviewed and approved by Sir Run Run Shaw Hospital.

## Author Contributions

Conceptualization: YZ and JC. Methodology: YZ, JC, and ZH. Investigation: ZH, HP, XS, and WC. Visualization: QM, TL, ZH, and QC. Supervision: YL, QM, and CG. Writing – original draft: YZ, JC, and ZH. Writing – review and editing: YZ, ZH, YL, and QM. All authors contributed to the article and approved the submitted version.

## Funding

This work was supported by the Zhejiang Provincial Natural Science Foundation (Y22H039489, LQ19H160044) and Zhejiang Province Medical and public health projects(2022519993, 2022522045).

## Conflict of Interest

The authors declare that the research was conducted in the absence of any commercial or financial relationships that could be construed as a potential conflict of interest.

## Publisher’s Note

All claims expressed in this article are solely those of the authors and do not necessarily represent those of their affiliated organizations, or those of the publisher, the editors and the reviewers. Any product that may be evaluated in this article, or claim that may be made by its manufacturer, is not guaranteed or endorsed by the publisher.
