# Supplementary material for: Platelet-Vesicles-Encapsulated RSL-3 Enable Anti-Angiogenesis and Induce Ferroptosis to Inhibit Pancreatic Cancer Progress
Source: Front Endocrinol (Lausanne). 2022 Mar 24;13:865655. doi: 10.3389/fendo.2022.865655 (PMC8987003; doi:10.3389/fendo.2022.865655)
Supplement: Supplementary file 1 [file DataSheet_1.docx]

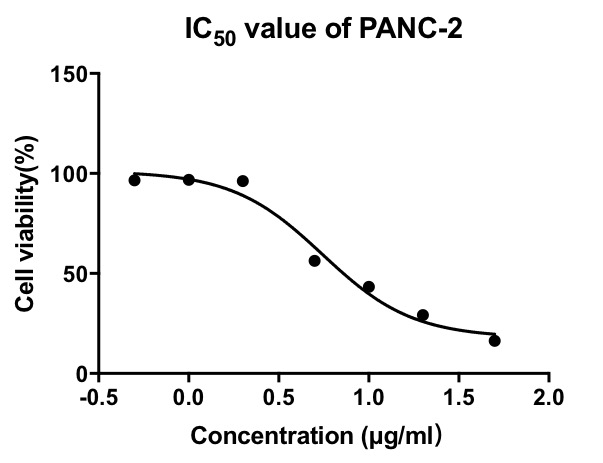


**Figure S1. The IC_50_ value of PANC-2 cell line.**


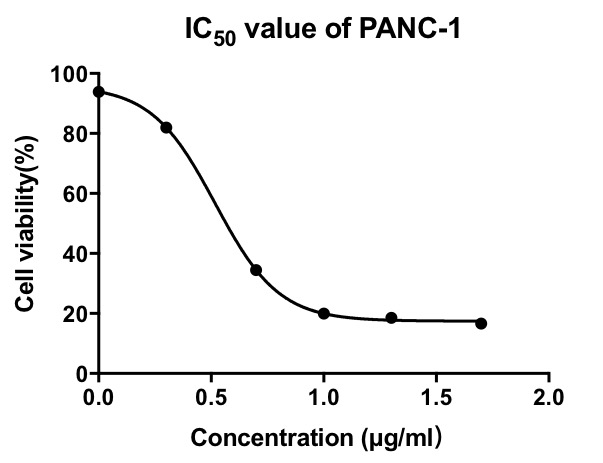


**Figure S2. The IC_50_ value of PANC-1 cell line.**


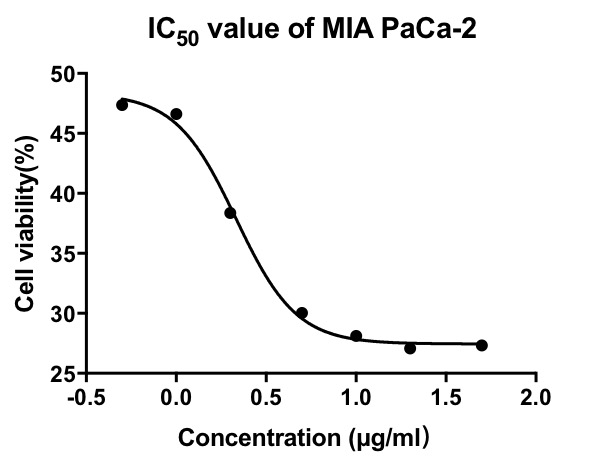


**Figure S3. The IC_50_ value of MIA PaCa-2 cell line.**

**Supplementary table 1.** Detail information for the primers we used in our study. F, forward; R: reverse.

| Gene names | Sequences (5′-3′) |
| --- | --- |
| GPX4-F | GAGGCAAGACCGAAGTAAACTAC |
| GPX4-R | CCGAACTGGTTACACGGGAA |
| M-GPX4-F | GATGGAGCCCATTCCTGAACC |
| M-GPX4-R | CCCTGTACTTATCCAGGCAGA |
| PTGS2-F | TTCAACACACTCTATCACTGGC |
| PTGS2-R | AGAAGCGTTTGCGGTACTCAT |
| M-ASLC4-F | CTCACCATTATATTGCTGCCTGT |
| M-ASLC4-R | TCTCTTTGCCATAGCGTTTTTCT |
| SLC7A11-F | TCTCCAAAGGAGGTTACCTGC |
| SLC7A11-R | AGACTCCCCTCAGTAAAGTGAC |
| M-SLC7A11-F | GGCACCGTCATCGGATCAG |
| M-SLC7A11-R | CTCCACAGGCAGACCAGAAAA |
| CD31-F | AACAGTGTTGACATGAAGAGCC |
| CD31-R | TGTAAAACAGCACGTCATCCTT |
| M-CD31-F | ACGCTGGTGCTCTATGCAAG |
| M-CD31-R | TCAGTTGCTGCCCATTCATCA |
| GAPDH-F | GGAGCGAGATCCCTCCAAAAT |
| GAPDH-R | GGCTGTTGTCATACTTCTCATGG |
| M-GAPDH-F | AGGTCGGTGTGAACGGATTTG |
| M-GAPDH-R | TGTAGACCATGTAGTTGAGGTCA |
